# *E2F1 *and *KIAA0191 *expression predicts breast cancer patient survival

**DOI:** 10.1186/1756-0500-4-95

**Published:** 2011-03-31

**Authors:** Robin M Hallett, John A Hassell

**Affiliations:** 1 Department of Biochemistry and Biomedical Sciences, Centre for Functional Genomics, McMaster University, 1200 Main Street West, Hamilton, Ontario, L8N 3Z5, Canada

## Abstract

**Background:**

Gene expression profiling of human breast tumors has uncovered several molecular signatures that can divide breast cancer patients into good and poor outcome groups. However, these signatures typically comprise many genes (~50-100), and the prognostic tests associated with identifying these signatures in patient tumor specimens require complicated methods, which are not routinely available in most hospital pathology laboratories, thus limiting their use. Hence, there is a need for more practical methods to predict patient survival.

**Methods:**

We modified a feature selection algorithm and used survival analysis to derive a 2-gene signature that accurately predicts breast cancer patient survival.

**Results:**

We developed a tree based decision method that segregated patients into various risk groups using *KIAA0191 *expression in the context of *E2F1 *expression levels. This approach led to highly accurate survival predictions in a large cohort of breast cancer patients using only a 2-gene signature.

**Conclusions:**

Our observations suggest a possible relationship between *E2F1 *and *KIAA0191 *expression that is relevant to the pathogenesis of breast cancer. Furthermore, our findings raise the prospect that the practicality of patient prognosis methods may be improved by reducing the number of genes required for analysis. Indeed, our *E2F1/KIAA0191 *2-gene signature would be highly amenable for an immunohistochemistry based test, which is commonly used in hospital laboratories.

## Background

Traditionally, a variety of clinical and histopathological characteristics have been employed to make predictions regarding the potential clinical outcomes of breast cancer patients. However, the advent of gene expression profiling technologies has enabled the use of molecular signatures to provide improved predictions of clinical outcome over traditional methods [1-5]. These signatures typically comprise many genes and require profiling their expression by measuring the abundance of their respective mRNA transcripts [3-5]. A major issue concerning the use of molecular signatures to provide prognostic information for cancer patients, is that transcript profiling tests require personnel with specialized training, as well as expensive reagents and equipment. These platforms are not routinely available in hospital pathology laboratories, which necessitates shipping tumor samples to an appropriately equipped laboratory, thereby increasing the time and cost of carrying out these tests. We hypothesize that identifying gene signatures that comprise 2-3 genes would enable the development of highly practical immunohistochemical based tests, which are commonly used in hospital based pathology laboratories.

Because the expression of proliferation associated genes has been shown to group breast cancer patients into good and poor risk groups [1], we sought to identify genes whose expression could increase the predictive accuracy of the proliferation gene, E2F1. E2F1 encodes a transcription factor that regulates the expression of target genes whose products participate in numerous processes such as DNA replication, mitotic check point, mitosis, DNA damage checkpoints, and DNA repair [6-8]. Generally, E2F1 is bound to and functionally inactivated by pRB; however, proliferative signals induce the phosphorylation of pRB by cyclinD/CDK4/6 complexes leading to the dissociation of pRB from E2F1, and the subsequent activation of E2F1 target genes [7]. In line with these observations, over-expression of E2F1 or various other members of the E2F gene family forces the re-entry of quiescent cells into S phase [9].

Using an algorithm we published recently [10], we found that the expression of *KIAA0191 *transcripts can be used in conjunction with those of *E2F1 *to more accurately predict breast cancer patient survival than does *E2F1 *expression alone. *KIAA0191*, commonly known as *TUT4 *or *ZCCHC11*, encodes a canonical poly (A) polymerase, whose function involves the polyadenylation of pre-mRNA in the nucleus [11]*. KIAA0191 *has been shown to work in concert with *Lin28 *to suppress microRNA biogenesis through uridylation of pre-microRNA. Importantly, *KIAA0191 *function has not been previously linked to *E2F1*. Here we demonstrate that the expression of *KIAA0191 *transcripts alone is not related to breast cancer patient survival. However, in the context of average to high expression of *E2F1 *transcript levels, high *KIAA0191 *expression was linked to poor breast cancer patient prognosis, whereas low *KIAA0191 *expression was linked to good outcome for these patients. Interestingly, our study identified a potentially novel functional relationship between *E2F1 *and *KIAA0191*, which may be of clinical relevance to breast cancer patients.

## Methods

### Microarray and clinical data

We used data from the Stanford microarray repository (downloaded from http://microarray-pubs.stanford.edu/wound_NKI/explore.html) for our analyses. We also downloaded a matrix containing clinical data for the patients that provided samples for the microarray profiles used in the present study from the same location. We created a master data matrix by combining the gene expression profiles with indices for survival and metastasis for each patient. Patients included within this cohort had either stage I or II breast cancer and were less than 53 years of age. The prevalence of lymph-node positive and lymph-node negative disease was approximately 50% for each, respectively.

### Identification of genes that enhance the predictive power of *E2F1*

To discover genes that might improve the capacity of *E2F1 *transcript levels to predict the prognosis of human breast cancer patients, we first ranked the level of gene expression for each gene in every patient's breast tumor as described previously[10]. We then adapted a similar approach to that we used previously, but instead of searching for genes whose expression was related to patient survival [10], we modified the algorithm to search for genes whose expression was predictive of patient survival in combination with that of E2F1. We then ranked all the genes present in the expression profiles using a scoring technique published previously [10].

### Survival and statistical analysis

Unless otherwise indicated all survival analyses and associated statistical tests were completed using GraphPad Prism 5™ software. Harrell's concordance-index (C-index) was calculated using the Hmisc package in R [12].

### Selection of random genes

Randomly selected genes were obtained by using a random number generator (http://www.random.org).

## Results

### *E2F1 *expression accurately groups patients into good and poor outcome groups

We sought to improve the capacity of a small number of genes to correctly divide breast cancer patients into good and poor prognosis groups. We started with a candidate gene approach, a methodology used in previous studies [4]. We chose to begin with *E2F1*, as its transcript levels are reportedly prognostic in human breast cancer [13], and because the E2F1 protein stimulates tumor cell proliferation, a process that is inversely correlated with breast cancer patient survival [6,8,14-16]. We also imagined that genes whose expression enhanced the prognostic power of *E2F1 *transcript levels to predict patient survival, might uncover genes whose products interacted directly or indirectly with E2F1.

**Figure 1:**
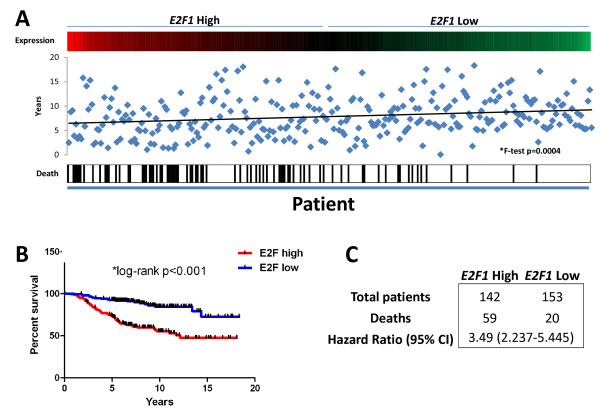
**E2F1 expression groups patients into good and poor overall survival groups**. A) Selection for the E2F1 high expression group and the E2F1 low expression group. B & C) Differences in endpoint between patients in the E2F1 high and low expression groups, where high E2F1 expression correlated with poor overall patient survival and low E2F1 expression was associated with a better overall patient survival.

To verify that *E2F1 *expression correlated with patient survival in large microarray breast cancer datasets, we made use of a database comprising a cohort of 295 breast cancer patients, whose tumors' gene expression profiles are known and for which clinical follow up data is available [2]. We first divided these patients into *E2F1 *high and low expressing groups by calculating the average expression of *E2F1 *transcripts in the tumors of all 295 patients, and used the average expression value to divide patients into high and low *E2F1 *expression groups. The latter process led to assignment of 142 patients to the *E2F1 *high expression group, and 153 patients to the *E2F1 *low expression group (Figure 1A). We considered overall survival as the endpoint for our analyses. We next measured the differences in endpoint between patients in the *E2F1 *high and low expressing groups, and found that high *E2F1 *transcript levels correlated with poor overall patient survival, whereas low *E2F1 *transcript abundance was associated with a better overall patient survival (Figure 1B, Log-rank, *p < 0.001, Figure 1C, Hazard Ratio (HR): 3.49 (2.237-5.445)).

Because *E2F1 *transcript abundance alone was not completely accurate at classifying patients into good and poor prognosis groups, we sought to identify other genes whose expression could augment the predictive power of *E2F1 *transcript levels. We first defined high, average, and low *E2F1 *expression based on expression above, within, or below the 95% confidence interval for *E2F1 *expression among all 295 patients. We then took a modified approach from that which we developed previously [10] to find genes that were i) generally highly expressed in tumors where high to average *E2F1 *expression was indicative of poor patient survival, and ii) generally were expressed at low levels in tumors where high-average *E2F1 *expression was not associated with poor patient survival. The mostly highly ranked candidate among the 295 patient cohort was *KIAA0191*, which is also commonly known as *TUT4 *or *ZCCHC11 *[11].

**Figure 2:**
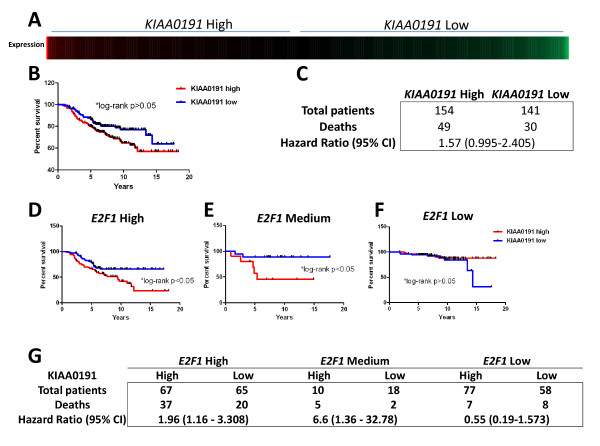
**KIAA0191 expression is not prognostic when used alone, but is prognostic in the context of average to high E2F1 expression**. A) KIAA0191 expression for all patients B & C) Survival comparison between the KIAA0191 high expression group and the KIAA0191 low expression group; there was no survival difference between the KIAA0191 high and low E2F1 expressing groups. D-G) KIAA0191 expression in relation to patient survival in the context of specific levels of E2F1 expression. Patient population divided into E2F1 high, average, and low expression groups, then KIAA0191 expression was measured and related to patient survival in each group.

To learn whether *KIAA0191 *expression alone was related to patient survival, we divided the patient cohort into *KIAA0191 *high and low expressing groups, as described above. This led to 154 patients being selected for the *KIAA0191 *high expression group and 141 patients being selected for the low expression group (Figure 2A). We compared survival between these two groups and found that there was no statistically significant difference in survival between the *KIAA0191 *high and low expressing groups (Figure 2B, Log-rank, p > 0.05, Figure 1C, HR: 1.57 (0.995-2.405)). We next determined whether *KIAA0191 *transcript levels were related to patient survival in the context of specific levels of *E2F1 *expression. We divided the 295 patient cohort into *E2F1 *high, average, and low expression groups as described above. We then determined whether *KIAA0191 *expression was related to patient survival in various *E2F1 *expression level subgroups. We stratified the patients within each *E2F1 *expression subgroup on the basis of high and low *KIAA0191 *expression, and compared survival of these patients. *KIAA0191 *transcript levels were related to patient survival in the context of high and average *E2F1 *expression, but not low *E2F1 *expression (Figure 2D-G, *E2F1 *high: Log-rank, *p < 0.05, HR: 1.96 [1.16-3.308], *E2F1 *Medium: Log-rank, *p < 0.05, HR: 6.6 [1.36-32.78], *E2F1 *Low: Log-rank, p > 0.05 HR: 0.55 [0.19-1.573]).

### Integration of *KIAA0191 *into *E2F1 *expression based prognosis decision-making

**Figure 3:**
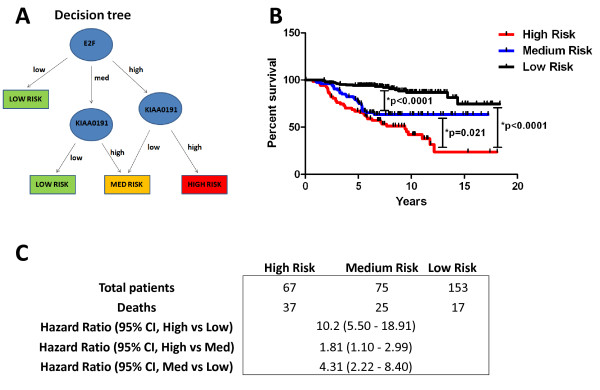
**KIAA0191 and E2F1 expression can be combined to accurately group patients into low, medium and high risk groups**. A) Tree-based decision strategy to integrate KIAA0191 expression into our model of using E2F1 expression to separate breast cancer patients into good and poor prognosis groups. B) % survival over time for low, medium and high risk patients. Patients with low E2F1 expression group directly into the low risk group, however, when patients had either high or average E2F1 expression we also used KIAA0191 expression to classify these patients among the various risk groups. Patients with average E2F1 expression and either low or high KIAA0191 expression where grouped into low and medium risk groups, respectively, whereas patients with high E2F1 expression and either low or high KIAA0191 expression were grouped into medium and high risk groups. C) Patients who survived or succumbed to breast cancer for each risk group, as well as associated hazard ratios between risk groups.

Because we found that *KIAA0191 *expression was only predictive of patient survival in the context of E2F1 transcript levels, we devised a tree-based decision strategy to integrate *KIAA0191 *expression into our model of using *E2F1 *transcript abundance to separate breast cancer patients into good and poor prognosis groups (Figure 3A). Because *KIAA0191 *expression was not linked to patient survival in *E2F1 *low expressing patients, and these patients had good overall survival, we grouped patients with low *E2F1 *expression directly into the low risk group (Figure 3B&3C, LOW RISK: Log-rank [High vs Low], *p < 0.0001 HR: 10.2 [5.50-18.91], Log-rank [Med vs Low], *p < 0.0001 HR: 4.31 [2.22-8.40]). However, when the patient's tumor expressed either high or average *E2F1 *transcript levels, we also used *KIAA0191 *expression levels to classify these patients among the various risk groups. Patients whose tumors expressed *E2F1 *transcripts of average abundance and transcripts of *KIAA0191 *at low or high levels where divided into low or medium risk groups, respectively, whereas patients whose tumors expressed *E2F1 *transcripts at high levels and that of *K1AA0191 *at either low or high *KIAA0191 *levels were grouped into medium and high risk groups, respectively (Figure 3B&3C, MEDIUM RISK: Log-rank [High vs Med], *p = 0.021 HR: 1.81 [1.10-2.99], Log-rank [Med vs Low], *p < 0.0001, HR: 4.31 [2.22-8.40], HIGH RISK: Log-rank [High vs Low], *p < 0.0001 HR: 10.2 [5.50-18.91]. To assess the predictive accuracy of E2F1/KIAA0191, we calculated Harrell's C-index [12]. In this fashion, a C-index value of 0.5 indicates predictive performance which is no better than chance, whereas values greater than 0.5 indicate true predictive capacity. We calculated Harrell's C-index for two different comparisons, where we compared predicted high risk patients to predicted low risk patients (Harrell's C-index: 0.75), and predicted high and medium risk patients to predicted low risk patients (Harrell's C-index: 0.71). In both cases, the C-index values were greater than 0.5 indicating true predictive performance of our E2F1/KIAA0191 signature.

**Figure 4:**
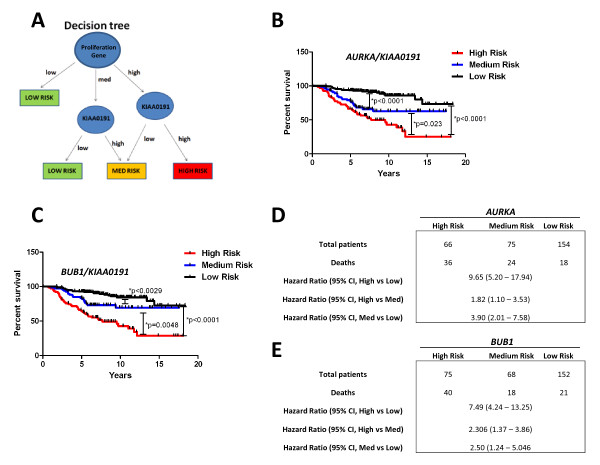
**KIAA0191 expression is also prognostic in the context of AurKA expression**. A) Tree-based decision strategy to integrate KIAA0191 expression into our model using the expression of a proliferation gene to separate breast cancer patients into good and poor prognosis groups. B & C) % Survival over time for low, medium and high risk patient groups, as well as D & E) associated hazard ratios between risk groups.

We also tested whether *KIAA0191 *expression was prognostic in the context of the expression 2 other genes, *Aurora kinase A *(*AURKA*) and *BUB1*, which like *E2F1*, are independently prognostic (data not shown) and linked to cell proliferation [17-20]. We found that grouping patients into high, medium and low risk groups (as described above), by interchanging either *AURKA *or *BUB1 *transcript levels for that of *E2F1*, resulted in highly similar risk grouping as observed with *E2F1 *expression (Figure 4A-E). Taken together, these results suggest that the relationship observed between KIAA0191 and E2F1 is also shared between KIAA0191 and other proliferation-associated genes.

## Discussion

Prognostic tests, which identify high and low risk cases of breast cancer, are greatly beneficial for identifying patients who can be spared unnecessary chemotherapy. For example, several clinical trials, including the National Surgical Adjuvant Breast and Bowel Project trials B-14 and B-20, have shown that adding chemotherapy to tamoxifen treatment increases survival in node-negative, estrogen-receptor-positive breast cancer patients [21-23]. However, the 10 year recurrence rate with tamoxifen treatment alone is only 15%, therefore if all patients were to equally receive additional chemotherapy, it would result in 85% of patients receiving little chemotherapy-derived benefit but nonetheless suffering its deleterious side effects.

In attempt to spare patients unnecessary chemotherapy, treatment decisions have traditionally been made based primarily on classical histopathological and immunohistochemical techniques. However, within the last several years, many genomic based molecular signatures have been derived that correlate gene expression in tumor tissue to breast cancer recurrence [2-5]. Importantly, many of these gene signatures more accurately assign risk to breast cancer patients than conventional criteria. However, a practical limitation of these signatures is that assays of transcript abundance require relatively intact RNA, as well as expensive equipment and technical expertise, which is unavailable in most hospital pathology laboratories. Hence, tumor specimens are commonly shipped to specialized clinical laboratories thereby increasing the turn-around time and cost of these tests. For these reasons, we sought to determine whether we could generate relatively small gene signatures (2-3 genes), which might yield accurate prognostic information. Indeed, a signature comprising 2-3 genes might be developed into an immunohistochemistry assay, which could be carried out in hospital-based pathology laboratories thereby saving both time and cost.

We began our experiments by choosing a single gene using a candidate gene approach. Because tumor cell proliferation is linked to poor survival in breast cancer patients, we first tested whether the expression of the single "proliferation" gene, *E2F1*, was also linked to survival in breast cancer patients [5,6,8,9,15,16,18]. The observation that high expression of *E2F1 *transcripts indicated poor overall patient survival in the dataset used for this study is unsurprising, given that tumor cell proliferation is associated with poor patient survival in other large breast cancer patient datasets, and low *E2F1 *transcript levels have previously been linked to good patient survival [13,18].

We next sought to identify additional genes whose expression might augment the predictive accuracy of *E2F1 *expression such that a highly accurate 2-gene signature might be developed. Indeed, such genes would be useful for increasing the accuracy of genomic based clinical outcome predictors, as well as understanding *E2F1 *based proliferation programs in breast cancer cells. Our analyses revealed that *KIAA0191 *transcript abundance could be used in the context of average to high levels of *E2F1 *transcripts to more precisely predict breast cancer patient survival. However, in the context of low *E2F1 *transcript levels, *KIAA0191 *expression was not linked to patient outcome. These results suggest that there is a relationship between *E2F1 *and *KIAA0191 *expression, which is predictive of patient outcome, and that there is a likely complementary involvement of both genes in breast cancer progression. Importantly, the observation that the expression of other proliferation genes, such as *AURKA *[18,20], *BUB1 *[17,19,24] could be used to replace *E2F1*, suggests that the relationship of *KIAA0191 *expression to patient survival is linked to cell proliferation. Indeed, these observations highlight that there is a potentially novel functional relationship between cell proliferation and KIAA0191. Indeed, this relationship appears to be important for the pathogenesis of breast cancer and is a topic that warrants further investigation.

Using the data available for this study it wasn't possible to measure the exact predictive accuracy of our 2-gene signature in an unbiased manner. From our initial analyses the predictive power of the E2F1/KIAA0191 2-gene signature looks quite promising (High vs Low, HR: 10.2 [5.5-18.9], Harrell's C-index: 0.75, Medium vs Low, HR: 4.3 [2.2-8.4], Harrell's C-index: 0.71), however future studies will need to replicate these findings using independent gene expression data sets.

An advantage of our 2-gene signature over currently available prognostic signatures is that it may be suitable for development as an immunohistochemical based test. As mentioned previously, immunohistochemical based tests are faster, cheaper, and have greater availability to patients, than the currently available mRNA based tests. Furthermore, antibodies that `recognize E2F1 and KIAA0191 are commercially available, and several protocols exist for the quantification of protein expression using immunohistochemistry [25]. However, there are significant differences in the technology platforms used for gene and protein expression assays (differences in dynamic range, linearity of relationship to clinical outcome), and therefore, genes which perform well using mRNA based expression profiling technology may/may not perform as well using a protein expression based immunohistochemical test [26]. Beyond this issue, the exact correlation between mRNA and protein expression remains poorly studied, although some initial work suggests that the correlation is significant [27]. As a result, it is important to note that this aspect of our study remains largely theoretical, as it is unclear how well such an immunohistochemical test would work for patient prognosis. To this end, validation of the 2-gene signatures using immunohistochemistry is a major focus of our current studies.

A major implication of this study is that it is important to understand the context in which a gene's expression is most highly related to patient survival. For example, we observed that high *E2F1 *expression was most related to poor patient outcome when that patient's tumor also expressed high levels of *KIAA0191*. When *KIAA0191 *was not expressed at high levels, the relationship between high *E2F1 *transcript levels and poor outcome was significantly reduced. In line with these observations, average levels of *E2F1 *expression were associated with poor patient outcome when *KIAA0191 *was highly expressed, and good patient outcome when *KIAA0191 *was expressed at low levels. Indeed, we took advantage of this relationship to generate a 2-gene based decision tree, which made highly accurate predictions about patient outcome, while only taking into account the expression of 2 genes.

## Conclusion

We envision that the identification of gene signatures, which are highly predictive, but consist of relatively few genes (2-3 genes), would allow the use of immunohistochemical or immunofluorescent based assays that are commonly used in hospital-based pathology laboratories to readily guide the use of chemotherapeutics in breast cancer patients. Importantly, immunohistochemical or immunofluorescent testing does not require long distance transfer of tumor samples to molecular profiling facilities (as is the case for MammaPrint™ and Oncotype DX) and thus would provide a less time-consuming and less costly means of providing prognostic information to breast cancer patients.

## Competing interests

The authors have filed a provisional patent on the use of 2-gene signatures for breast cancer patient prognosis.

## Authors' contributions

RMH, conception of project and performed research; RMH, and JAH, interpretation of data and writing of manuscript. All authors have read and approved the final manuscript.
